# PATIENT AND PHYSICIAN EXPOSURE TO X-RAYS AT PEDIATRIC INTERVENTIONAL CARDIOLOGY – FROM WORLD TO POLAND

**DOI:** 10.13075/ijomeh.1896.02504

**Published:** 2024

**Authors:** Mateusz Mirowski, Joanna Domienik-Andrzejewska, Tomasz Moszura

**Affiliations:** 1 Nofer Institute of Occupational Medicine, Radiation Protection Department, Łódź, Poland; 2 Polish Mother’s Memorial Research Institute, Department of Pediatric Cardiology, Łódź, Poland

**Keywords:** occupational exposure, optimization, ionizing radiation, pediatric cardiology, patient exposure, interventional cardiology

## Abstract

Ionizing radiation is considered as a harmful factor to health. However, X-rays are widely used in diagnostic and therapeutic procedures such as those performed during cardiac interventions. Their use is particularly invaluable in saving life procedures when the risk of adverse effects of radiation is relatively low compared to the consequences of non-treated or treated with other invasive methods diseases. One branch of these types of medical procedures is interventional cardiology in pediatrics due to possible higher than in adults risks of developing cancer in exposed to ionizing radiation children. On the other hand, medical staff in particular physician, may be exposed to relatively high radiation levels during their work. Parallel with enlarging workload (growing number of procedures per year), high levels of cumulative doses to most exposed, and often not protected, parts of operator’s body as eye lenses and brain may be achieved. Exposure to X-rays in pediatric interventional cardiology is a worldwide point of scientific interest from around 65 years, however assessment and simulating low level doses is still developing. In this review found data presents various trials of evaluating doses or levels of exposure to both medical staff and patient as well as methods of optimization and protection against X-rays in pediatric cardiac interventional procedures. The issue of establishing diagnostic reference levels was also taken into consideration during analysis. Thirty papers from period 2013 to 2023 were analyzed. The main assumption of this condensed review is to reveal radiation protection methods worldwide and in Poland.

## Highlights

- X-rays are widely used at medical diagnostics and therapy, however this radiation might be harmful.
- Growing number of interventional procedures conducted, where X-rays are in use, should be monitored in terms of exposure and risks, also in pediatric interventional cardiology.
- Consciousness and prevention is suggested, as well as multidisciplinary cooperation.

## INTRODUCTION

It is proven that ionizing radiation might be a harmful factor to health of human beings. We can list many types of radiation like corpuscular as α (particle of helium) or β (emitting electrons [–]/positrons [+]) or propagate as waves: γ (emitted mainly by isotopes) or X-rays (generally produced by stopping accelerated electrons in vacuum tube). The last one from above-mentioned types of ionizing radiation is constantly popular and its use is still being developed at various medicine fields such as interventional cardiology. Intravascular methods of diagnostics and therapy of cardiac and vascular malformations are also getting more popular (sometimes being a gold standard for medical procedure). These both elements − X-rays and interventional cardiology are oftentimes inseparable. Ionizing radiation is a phenomena, enabling operator to see the pathway and movement of, e.g., stent from the place of insertion to the target point of patient’s body. The apparatus which allows the medical staff to control the implementation and fulfilling the medical procedure is an intraoperative roentgen diagnostic device, so-called “C-arm.” Usage of this type of device is connected with radiation exposure − both for patient and medical staff (for patient, coronarography is comparable with, e.g., 1 chest [thorax]computed tomography [CT] which delivers to the whole body the effective dose [E] of around 6.5 mSv [1]). Operator, physician who conducts the procedure, may be exposed to large amounts of radiation from primary beam and scattered radiation despite using shields as lead or lead-free apron, leaded glasses and, e.g., ceiling suspended shield. Relatively high level of cumulative doses received by physicians is also caused by the workload − number of medical cases is still enlarging, as in Poland in 2009–2018 pediatric interventional catheterization cases has grown from 1221 to 2271 [2].

The issue of pediatric exposure to X-rays from fluoroscopy-guided procedures is considered from more than half of the century [3]. Pediatric interventional cardiology is a branch of medicine, specialized to diagnose and treating of congenital heart defects (CHD) in young patient heart and near arteries. It is known that slightly >1% of live births may characterize cardiac malformations [4]. In the European Union (EU), 3 879 509 live births were recorded in 2022, of which Poland’s contribution was 305 132 newborns [5,6]. Statistically it gives circa 3051 cases/year in Poland [7].

Moreover, mortality caused by CHD is declining with growing socio-demographic index [8]. Diagnostics and therapeutic interventions may be conducted parallel to surgical treatment. Hence, it is understood that young patients from newborn to maturity might undergo several treatments and therefore be exposed to high levels of radiation during this period. Being aware of high radiosensitivity due to cellular activity (Bergonie and Tribondeau law) connected to growing human body, long life expectation and closer distance to primary beam of all organs during interventional diagnostics or treatment, an assessment of radiation exposure and its optimisation have to be a priority. Radiation protection of patient should be scrutinized by specialists and scientists, especially physicians, medical physicists and manufacturers or providers of the imaging equipment. According to UNSCEAR report [1], knowledge about cancer risk of pediatric patient’s group is not wide, however it might be a 2 or 3 times higher than for whole group of all ages. Moreover, exposure during cardiovascular procedure is not the only one type of irradiation during a single treatment of CHD. It might be also accompanied with planar radiography, CT or imaging using nuclear medicine.

The aim of this article is to show the condensed review of patients and physicians exposure during cardiac pediatric interventions and its optimisation. The review is mainly based on papers and reports from the United States of America (USA), Canada and the EU including Poland.

## METHODS

Mainly, articles have been searched using 2 types of scientific databases: Science Direct and Springer. There were parallel used keywords:“interventional cardiology,” “dose” and “pediatric.” Obviously, from the group of found manuscripts, only those related to pediatric cardiovascular interventions were selected. Lots of articles were wrongly suggested/matched by browser (e.g., articles were about usage of magnetic resonance imaging [MRI], or dosing medicines in pediatric cardiology). What is more, Google Scholar has been used. Summing up, after rejection of articles older than 10 years (not being published during period of years 2013–2023) 30 papers about pediatric interventions were used (with 15 additional publications, reports and links, essential to prepare consistent contents).

## RESULTS

### Exposure of young patients during interventional cardiology procedures

A number of researches and reviews from the period 2013–2023 concerning the radiation exposure of children during interventional cardiology have been found. Baysson et al. [9] prepared French cohort of children (permanent citizens of France) who undergone cardiac catheterization since 2000 and also were <10 years old. Retrospective analysis from electronic patient datasets (having parameters of body and technical environment) was used to estimate radiation exposure. This database, which was expected at the time of publishing to collect 8000 patients, might be useful for further analysis of cancer risks in pediatric interventional cardiology exposure. Moreover, a few authors of the previous article were involved in 2 other researches [10,11] on establishing local diagnostic reference levels (DRLs) and assessing organ doses in pediatric interventional procedures. Using dosimetric parameters such as dose area product (DAP), time of fluoroscopy and number of cine frames, and taking into consideration patients’ body weight, as well as physical measurements with thermoluminescent dosimeters and anthropomorphic phantoms in conjunction with virtual environment for Monte Carlo methods simulations, Barnaoui et al. [10] analyzed exposure and assessed doses for lungs, esophagus, breast, thyroid and E to patient. They grouped gathered data from one of French reference centre (2010–2011) into diagnostic and therapeutic procedures. For first type, the evaluated E was 0.3–23 mSv (M = 4.8 mSv) while for second − 0.1–48.4 mSv (M = 7.3 mSv). Organ doses to newborns’ lungs and oesophagus were indicated as the most prominent. In terms of procedure type − angioplasty was assessed as with the highest doses 0.6–48.4 mSv (M = 13 mSv). The authors suggest to establish diagnostic reference level for pediatrics in terms of increasing use of using interventional procedures in above-mentioned cases. Second paper [11] treats the risk of cancer induction in children who have undergone pediatric interventional procedures. The authors take on an assessment of 1251 pediatric procedures conducted in 2 reference centres in France (2009–2013). Data of 1251 procedure on children ≥15 year old was grouped by type of procedure (atrial septal defect [ASD] closure, patent ductus arteriosus [PDA] occlusion, pulmonary valvuloplasty [PV]) and gender specific lifetime attributable risks (LAR) were projected. For around 10% of all types of procedures with higher exposure, LAR reached 4.2/1000. The authors conclude that in some cases doses may be prominent and there is an increase of cancer risk, so dose reports are suggested to be done.

In addition, Johnson et al. [12] analyzed group of children (N = 337) with age ≥6 years who undergone in the past 1 from 7 typical interventions, e.g., connected with ASD. Moreover, other types of imaging were analyzed (conventional radiography and CT). Data was collected in 1 medical institution during period of 2005–2010. Research shows that median cumulative E was at level of 2.7 mSv and median LAR of cancer dependson complexity of medical procedure and ranges 0.006–1.6%. As the conclusion, the authors say that radiation exposure to patient from all modalities is relatively low, however, selected group was exposed to significant amounts of radiation. What is more, cancer risk estimation shows the necessity of limiting doses, mainly in case of modalities with high exposure.

Another retrospective research was conducted in the Philadelphia, USA, where Glatz et al. [13] reviewed all pediatric cases performed in 2009–2011, using one C-arm unit (with exclusion of electrophysiology cases). In this study, Monte Carlo methods were used to calculate E in mSv from DAP in μGy × m^2^. For 2265 cases median DAP was 760 μGy × m^2^ and assessed median E was 6.2 mSv. The authors concluded that radiation monitoring is of great importance at pediatric interventions and results in further strategies of dose reduction.

Ghelani et al. [14] published in 2014 a paper treating of benchmarking radiation dose levels, with correlation to age of patient suffering on CHD and types of conducted procedures. Data collected from 7 laboratories include total air kerma, DAP, and total fluoroscopy time. It was divided into 6 groups of medical procedures: PDA, ASD closure, PV, aortic valvuloplasty (AV), treatment of coarctation of aorta (CoA) and transcatheter pulmonary valve (TPV) replacement. Finally, 2713 cases were collected from years 2009–2013. The benchmark was prepared as a collection of median and 75th and 95th percentiles. Apart from dose assessment (which was extended and worth to study in original paper), as the additional outcome, it was concluded that standing alone, the fluoroscopic time is inappropriate to monitor radiation exposure. Moreover, it is suggested that the collected data might be a baseline to Congenital Cardiac Catheterization Project on Outcomes (C3PO), conducted in USA [14]. In terms of C3PO Quality Improvement initiative, Quinn et al. [15] proposed diversification of patients with CHD into categories with similar radiation exposure. Collected data from period between whole 2014 and 2015 was used (9 centres from C3PO). Total number of 11 735 cases was categorized into low, medium and high exposure. Ratio between DAP and patient mass was assessed. Value <100 is treated as low exposure, 100 to <200 as medium, and ≥200 as a high exposure. Number of cases for each group and median DAP/kg stands as follows: 7918 and 39, 1807 and 131, 11 and 231, respectively. The authors found this as a huge step towards radiation exposure optimization for CHD cases.

In Canada, likewise to Johnson et al. [12], Walsh et al. [16] had a trial to assess cumulative radiation exposure, taking into account doses from not only fluoroscopic guided interventions. The yield of CT scans and other modalities to calculate final doses during treatment of CHD were also included. The study comprised 70 patients subjected to Fontan surgery, whose mean age was 3.6 years. It was found that the mean number of chest X-rays was 32 (cumulative exposure M = 1320 μGy × m^2^/patient) and the mean number of cardiac catheterization was 2.45 (fluoroscopy exposure M = 1103 μGy × m^2^/case, cineangiography exposure M = 1412 μGy × m^2^/patient). To sum up, according to the authors, it gives a cumulative exposure of M = 9054 μGy × m^2^/patient from whole intervention, considering fluoroscopy and cineangiography. Furthermore, M = 0.44 CT scans/patient were made, with an ex posure of M = 154 μGy × m^2^/person. Mean cumulative exposure from planar radiography during pediatric cardiac examinations was about 1320 μGy × m^2^/patient. Above-mentioned data shows that young patients might have a high cumulative dose during treatment of CHD. Walsh et al. suggested to consider usage of other non-ionizing modalities as MRI if it is possible.

The year 2014 seems to be of high interest concerning the assessment of cumulative dose in pediatric cardiac interventions. In this year, Brambilla et al. [17] published a document − a systematic review about exposure to ionizing radiation in non-oncologic chronic illnesses, including interventional cardiology. From variety of published information, it is seen that the cumulative E to CHD patients is 3.5–4.5 mSv for diagnostics, however, for therapeutics it might be about 6 mSv. Furthermore, Gould et al. [18] presented a systematic review of radiation doses exact in pediatric cardiac catheterisation. Data presented here considered: DAP, E, peak skin dose and organ doses; the mean and median values were reported. Mean values of DAP for diagnostics was 294–2088 cGy × cm^2^ and for interventional procedures 243–10 900 cGy × cm^2^. Median DAP varies in ranges 186–71 240 cGy × cm^2^ for diagnostics and 70–26 930 cGy × cm^2^ for interventions. Values of min.-max for mean E observations ranges 0.2–23.2 mSv and 0.3–48.4 mSv for diagnostics and therapeutics, respectively. For median observations, min.-max is denoted as between 0.16–27.8 mSv and 0.38–25.7 mSv for diagnostics and therapeutics, respectively. Mean peak skin dose varies 16–190 mGy taking into consideration diagnostics and therapeutics as one. For median, the same range is 23.9–140 mGy. Some of organ doses are presented in this review. Highest mean absorbed dose is seen to lungs (33.45 mGy), highest value from equivalent dose ranges is also for lungs (93.7 mSv). The most prominent mean equivalent dose is seen to thymus (122.5 mSv) [18].

Nicholson et al. [19] concluded in his paper that increased awareness of physicians to radiation exposure, results of the dose reduction to patient. In this research, collected data was divided into 3 eras: January 2009 − January 2011, January 2011 − September 2013 and September 2013 − May 2014. Across these eras the authors can see a decrease of cumulative air kerma measured in mGy as the years go by. The authors cannot explain the decrease between first and second eras, however they suggest that between second and third, there were implemented various strategies for dose reduction. The most important is decrease of frame rate during digital angiography.

From technical side related to the equipment being used, the American Association of Physicists in Medicine (AAPM) published in 2022 a report concerning the impact of patient size (from infant to adult size) simulated by polymethyl methacrylate (PMMA) phantoms on air kerma rate values in pediatric fluoroscopy [20]. The paper indicates that some of X-ray apparatuses may not be adjusted to pediatric standards. Configuration and design of the machine protocols should be suited for children procedures. As result, it should provide especially lessen the air kerma rates, which consequence is declining of exposure to X-rays. Survey shows that 80% of pediatric patients are treated using protocols suited to adult patients.

### Exposure of physician to X-rays during interventional cardiology procedures

Regarding physicians’ exposure working in interventional cardiology departments, in recent decade, the interest of researchers was focused on eye lenses exposure due to the >7-fold reduction of the annual limit to this organ (from 150 mSv to 20 mSv). In EU this new dose limit to the eyes was laid down in European directive 2013/59/Euratom [21]. In Poland, based on this new directive, there was an adjustment of the Polish Atomic Law in year 2019 [22]. With this respect, however, the most studies did not include pediatric interventional cardiology. The most important European projects concerning the assessment of eye lens doses to physicians performing IC procedures with adult patients were “Optimization of radiation protection of medical staff” (ORAMED), “The European epidemiological study on radiation-induced lens opacities among interventional cardiologists” (EURALOC) and “Implications of Medical Low Dose Radiation Exposure” (MEDIRAD) (all these projects were conducted also in Poland at Radiation Protection Department of Nofer Institute of Occupational Medicine in Łódź, Poland). The former one was launched to assess the levels of, previously not measured on the routine basis, doses to eye lenses (doses per procedure, annual doses) while EURALOC project to study the cumulative eye lens doses [23–25] and the prevalence of opacities among interventional cardiologists; the end point of the project was to analyze the dose–response relationship. In turn, in the last project, new protective tools (such as lead and non lead caps, masks, aprons and blankets as well as the zero gravity system) were tested for their effectiveness in reducing the doses, in particularly, to eye lens and brain [26–28].

One of these limited number of studies focused on eye lens exposure of physicians performing pediatric interventional cardiology procedures was conducted in Spain by Alejo et al. [29] who estimated maximum superficial eye lens doses in terms of Hp(0.07). Optically stimulated dosimeters (OSL) have been used and mounted on eyes position at anthropomorphic Alderson-Rando phantom’s head. The authors used distance of 60 cm between left eye and scattering PMMA phantom, being placed in primary beam of C-arm with AP projection. Parallel, there were assessed maximum annual dose to eye lens from data collected in pediatric interventions. From 325 studies collected in 2011–2012 there were obtained the third quartile of number of images and time of fluoroscopy. It was considered as the maximum workload and used to assess upper boundary of superficial eye lens dose. The maximal estimated superficial doses to eye in 2011 and 2012 were (with accordance to increasing workload): to the left eye − 9.8 mSv and 12.0 mSv and to the right eye − 8.7 mSv and 10.5 mSv, respectively. As a conclusion, there was no possibility to exceed newly established annual eye dose (or even the dose value classifying the worker into exposure category A) during Spanish pediatric cardiac procedures. In another article by Alejo et al. [30] describing the exposure of physician eyes during pediatric interventions the assessment was based on OSL dosimeters placed on leaded goggles. Also kerma area product (KAP) was gathered in this study. Doses were collected from 2 pediatric cardiologists, after 222 interventions conducted in 1 year. The eye lens doses were further correlated with whole body doses from the dosimeter worn over the apron. As results annual eye doses of 2 physicians were 4.13 mSv and 4.98 mSv. Doses over the apron were 10.83 mSv and 11.97 mSv, respectively. Correlation between superficial eye dose and “on apron” dose was R^2^ = 0.89 with ratio of 0.38. Normalized to KAP eye lens dose was estimated as 2.21 μSv/Gy × cm^2^. In conclusion, the authors state that personal dose equivalent is useful to estimate eye lens dose in case when no radiation protection device is used.

Ubeda et al. [31] evaluated entrance surface air kerma (ESAK), KAP and doses from scattered radiation during procedures in above-mentioned interventions. With usage of PMMA plates of different thickness (4–16 cm), simulating pediatric patient with age of 1–15 years and using also the protocols from 6 different hospitals in Chile − ESAK, KAP and eye doses from scattered radiation were calculated. Scattered dose to physician eye had been estimated in range of 0.2–116 μSv, differentiate to type of procedure and hospital. Scatter doses Hp(0.07) were estimated for ten procedures and 6 X-rays systems. With 4 thicknesses of PMMA phantom (4 cm, 8 cm, 12 cm, 16 cm), the highest doses were obviously seen with the thickest phantom and varies from 17–116 μSv for different systems [31].

### Methods of optimization the exposure during pediatric interventional cardiology

The main principle of optimization process in radiation protection is the ALARA (as low as reasonably achievable) which means that the ionizing radiation must provide more benefits than risks and harm for exposed people. The importance of optimization in X-ray usage at interventional cardiology is visible worldwide. In Canada, for example, Canadian Cardiovascular Society published in 2013 document [32] treating of recommendations about quality assuring in X-ray exposure. Paper was written about numerous types of cardiac interventions conducted in Canada, also about pediatric ones. Apart from aspects of pediatric exposure, previously mentioned in Introduction part, there is also added information about high heart rate, which implicates faster frame rates. Children with CHD undergo a lot of long-lasting and complicated procedures or 3D modelling of heart, what also results of higher exposure. The document describes also 2 campaigns: “Image Gently” and “Step Lightly” concerning methods of communication between medical staff and parents, in terms of risks during radiation-related procedures.

In case of 3D visualisation, Stelt et al. [33] presented condensed review of cases of pediatric patients who under went the cardiac interventions using 3 dimensional rotation angiography. Thirty-one cases have been shown including 4 from Poland: 3 conducted by Goreczny et al. (in 2016 and 2017, interventional, PDA closure, hypoplastic left heart syndrome (HLHS) ductal stenosis, percutaneous pulmonary valve implantation (PPVI), patient’s age 12 months, 20 days and 14 years, respectively) and 1 by Moszura et al. (in 2013, interventional, middle aortic syndrome, 3.5 years old patient). From 3 first cases, it is seen that DAP is strongly varying with highest total DAP 3D for youngest patient at level of 519.5 μGy × m^2^. In fourth case, DAP and other radiation values are not presented. The authors concluded that there are missing homogenous, in-depth researches performed in standardized manner to assess exposure. What is more, they mentioned that researches of 3D rotational angiography (3DRA) benefits should be also conducted. Minderhoud et al. [34] investigated a median E during 3DRA. Previously, they found median value 1.6 mSv. With using reduction pediatric protocol, the authors noticed 66% reduction of exposure using 3DRA and 79% in whole catheterization. Also, Stenger et al. [35] compared 3DRA to standard biplane imaging, in terms of assessment aortic diameter. In their paper [36] the authors compared DAP and air kerma, gathered from pediatric cathetereziation of 2 groups patient with weights <20 kg. Those children undergone PDA procedure with usage of standard imaging system and next-generation pediatric imaging system (based on air-gap technique). After data analysis, the authors observed 65–70% reduction of air kerma and DAP in second group, comparing both (with similar fluoroscopic time). Values of above mentioned data were 76 mGy to 28 mGy and 500 μGy × m^2^ to 199 μGy × m^2^, respectively.

### Establishing DRLs

As it was mentioned previously, pediatric exposure to X-rays during cardiac interventions should be of high concern. Nowadays, establishing local and national DRLs in pediatrics is being considered more solemn. In contrary to the occupational exposure, there are no limits to patient exposure. However, the optimization of patients’ doses is still needed and it is performed using DRLs for kerma in air or KAP. During selection of articles treating about DRLs, in period of years 2013–2023, 6 positions and 1 report have been found. One scientific research was conducted multinationaly in United Kingdom, Australia and Ireland [37]. In the EU, studies and trials of setting local DRLs were done in France [38], Greece [39], Belgium [40], Italy [41], and outside the Europe − in Chile [42]. These 6 papers present methods for evaluating and calculating DRL. The details are shown in Table 1.

**Table 1.** The annual changes of urban heat island (UHI) episodes in Łódź, Poland, 2014–2019

Reference	Country	Methods	Resultsand conclusions
McFadden et al. 2013 [37]	United Kingdom, Australia, Ireland	– doses and details of examinations recorded from 354 patients, using DAP, data categorised into 5 age groups (newborn, <1 year, 1 to <5 years, 5 to <10 years, 10 to <15 years, >15 years) and 2 groups: diagnostic/therapeutic	– patient age M = 2.6 years (min – max: 0 days – 16 years) – patient weight M = 14.9 kg (min – max: 2.4–112 kg) – local DRL for mentioned groups: 190 cGy × cm^2^, 421 cGy × cm^2^, 582 cGy × cm^2^, 1289 cGy × cm^2^, 1776 cGy × cm^2^
Barnaoui et al. 2013 [38]	France	– DAP, FT, number of cine frames analyzed with taking into account patient weight -(5 groups: <6.5 kg, 6.5–14.5 kg, 14.5–25.5 kg, 25.5–43.5 kg and >43.5 kg) – assessment of doses, effective dose and conversion coefficients using thermoluminescent dosimetry and simulations basing on Monte Carlo methods – groups: diagnostic and therapeutic	– diagnostic effective dose range 0.3–23 mSv (M = 4.8 mSv) – therapeutic effective dose range 0.1–48.4 mSv (M = 7.3 mSv) – highest values for angioplasty range 0.6–48.4 mSv (M = 7.3 mSv) – conclusion: patient exposure is relatively high, there is a need of setting DRLs and optimization
Kottou et al. 2018 [39]	Greece	– differentiation by interventional procedures and age, N = 710 patients (477 with -noted age) – age groups (<1 year, 1 to <5 years, 5 to <10 years, 10 to <16 years)	– values of FT (min), number of frames (n), KAP (Gy × cm^2^) for mentioned groups (5.8 min/1322/2.0 Gy × cm^2^, 6.5 min/1403/3.0 Gy × cm^2^, 5.9 min/950/7.0 Gy × cm^2^, 5.7 min/940/14.0 Gy × cm^2^) – for all patients ranges are: 3.1–15.8 min/579–1779/1.0–20.8 Gy × cm^2^ – conclusion: setting DRLs is challenging due to many clinical and technical factors
Buytaert et al. 2019 [40]	Belgium	– DRL calculated as 75th percentile of cumulative DAP values per procedure – simulations and assessments using MC software, DICOM RDSRs and Kruskal-Wallis H test	– strong correlation and narrow confidence between DAP and product of body weight multiplied FT – conclusion: 75th percentile of DAP normalized to body weight multiplied FT seems to be good DRL for all procedures
De Monte et al. 2020 [41]	Italy	– methods proposed by International Commission on Radiological Protection No. 135 and Radiation Protection No. 185 – patients selected by body weight; KAP and K_a,r_ were defined – group of 385 procedures – DRL-curve methodology has been used to assess relation KAP/weight	– the established DRLs were lower than published data – highest median values for angioplasty (4.9 Gy × cm^2^/11.6 Gy × cm^2^ for groups 5–15 kg/15–30 kg) – conclusion (LDRL) curve method promising for valvuloplasty and angioplasty – stratification by body weight not reducing variability of KAP
Ubeda et al. 2022 [42]	Chile	– doses from diagnostic and therapeutic cases were collected in 2020–2021 in Latin America – group of 968 procedures – 2 groups: diagnostic and therapeutic – differentiate to 4 age groups (< 1 year, 1 to <5 years, 5 to <10 years, 10 to <16 years) – differentiate to 5 weight bands (<5 kg, 5 to <15 kg, 15 to <30 kg, 30 to <50 kg and 50 to <80 kg)	– values obtained (diagnostics/therapeutic): for age groups: 2.9 Gy × cm^2^, 6.1 Gy × cm^2^, 8.8 Gy × cm^2^, 14.4 Gy × cm^2^/4.0 Gy × cm^2^, 5.0 Gy × cm^2^, 10.0 Gy × cm^2^, 38.1 Gy × cm^2^ for weight bands: 3.0 Gy × cm^2^, 4.5 Gy × cm^2^, 8.1 Gy × cm^2^, 9.2 Gy × cm^2^, 26.8 Gy × cm^2^/3.7 Gy × cm^2^, 4.3 Gy × cm^2^, 7.3 Gy × cm^2^, 16.1 Gy × cm^2^, 53.4 Gy × cm^2^

DAP – dose area product; DICOM – Digital Imaging and Communications in Medicine; DRL – diagnostic reference levels; FT – fluoroscopy time; K_a,r_ – air kerma at the patient entrance reference point;

KAP – kerma area product; LDRL – local diagnostic reference levels; RDSR – Radiation Dose Structured Report.

As it is seen, at the short period of time, different approaches for assessing DRLs were proposed. Problem of DRLs in pediatric interventional cardiology is still under investigation.

Moreover, the EU Report No. 185: “European Guidelines on Diagnostic Reference Levels for Pediatric Imaging” was published in 2018 [43]. In this online accessible manuscript, interested persons can learn about the suggested guidelines and read more extensive review of approaches in pediatric DRLs topic. There is, e.g., a 3-way approach of establishing DRLs (local, national and European) and the suggested relations between them [43].

In Poland, as a consequence of the implementation of the European directive 2013/59/Euratom to Polish Atomic Law, there is an obligation to use DRLs [44]. However, until the time of writing this review, DRLs for pediatric interventional cardiology are not assessed and published by local legislation [45].

## CONCLUSIONS

Interventional cardiology in pediatrics has a quite long history, however radiation protection and its optimization consciousness is still developing. In terms of epidemiologic studies cohorts are built for further analysis. In many papers, also some of analysis were recently done.

The authors are taking into account topics from air kerma measurements up to organ dose assessment with usage of Monte Carlo methods simulation software. Some of approaches were described in this paper (including data from hospitals, laboratory measurements basing on anthropomorphic phantoms etc.).

The authors of the cited papers suggest that there is a need of lowering exposure to X-rays by developing methods and apparatuses or exchanging X-rays to other ways of imaging such as MRI (if it is possible). Also, proper usage of radiation protection equipment is advised. When focusing on Poland, with context to outer countries and their activities, one can observe that there is also growing number of pediatric interventional procedures (with higher survivability). It causes one major implication − more prominent risk of occurrence of radiation induced illnesses for both: pediatric patient and occupational exposed medical staff. In terms of patient, there is still lack of DRLs for interventional cardiology in pediatrics. Concluding, we suggest to take special attention to above mentioned branch of medicine and radiation protection. Obviously, the highest priority should be put on conducting succeeded treatment of congenital heart disease/defect, however further health implications of X-rays usage to patient and medical staff are of high priority. Worth considering is: cooperation between physician, medical physicist, radiation protection officer, manufacturer etc. in Poland and worldwide, creating datasets of, e.g., past and further dose reports, analysis of data using newest IT tools as computer aided simulations or algorithms to inference and getting results. The work also indicates numerous topics of European research undertaken in adult interventional cardiology. In the authors’ opinion, similar studies and with at least similar intensity should be carried out in pediatric interventional cardiology in the group of patients who, due to their age, are particularly sensitive to ionizing radiation.
